# Efficacy and safety of dulaglutide compared with the first-line hypoglycemic drugs in Asian patients with type 2 diabetes: a systematic review and meta-analysis

**DOI:** 10.1038/s41598-022-22263-4

**Published:** 2022-10-31

**Authors:** Bin Yu, Fei Lin, Maoru Wang, Hong Ning, Baodong Ling, Youyi Rao

**Affiliations:** 1 Department of Pharmacy, Mianyang Central Hospital, Mianyang, People’s Republic of China; 2 Department of Pharmacy, The First Affiliated Hospital of Chengdu Medical College, No 278 Baoguang Avenue, Xindu District, Chengdu, 610500 People’s Republic of China; 3 Sichuan Province College Key Laboratory of Structure-Specific Small Molecule Drugs, Chengdu Medical College, Chengdu, People’s Republic of China; 4 Department of Drug Dispensing, The Third Hospital of Mianyang, Mianyang, People’s Republic of China

**Keywords:** Diseases, Endocrinology, Medical research

## Abstract

To assess the efficacy and safety of dulaglutide in the treatment of Asian type 2 diabetes mellitus (T2DM), along with first-line hypoglycemic drugs. Systematic review and meta-analysis. Cochrane Library, Pubmed, Embase, and www.clinicaltrials.gov databases were searched from inception to September 27, 2022. The studies evaluating adults (≥ 18 years) undergoing dulaglutide (0.75 mg and 1.5 mg) and first-line hypoglycemic drugs were considered. There were only English languages. We used Stata 12.0 software to detect the risk of bias. 4 randomized controlled trials (RCTs), and 1 observational study. Both dulaglutide 0.75 mg dose group and 1.5 mg dose group could significantly reduce HbA1c [Dulaglutide 0.75 mg: WMD = − 0.20, 95% CI (− 0.28, − 0.11), *P* < 0.0001; Dulaglutide 1.5 mg: WMD = − 0.49, 95% CI (− 0.67, − 0.30), *P* < 0.0001] in Asian T2DM patients. In reducing fasting blood glucose (FBG) level, there was no significant difference observed in 2 dose groups. The body weight of patients in both dulaglutide dose groups was significantly reduced. In safety, the incidence of adverse events in the dulaglutide 0.75 mg dose group was slightly higher than that in the first-line drug group, but there was no statistically significant difference in the incidence of adverse events between the 1.5 mg dose group and the first-line drug group. Furthermore, the incidences of hypoglycemic events in both groups were higher than that in the first-line drug group. Two doses of dulaglutide showed better efficacy for Asian T2DM patients, but patients should be vigilant about the occurrence of hypoglycemia and gastrointestinal discomfort. However, more number and better quality of RCTs are suggested to confirm long-term safety and efficacy.

## Introduction

The incidence of diabetes mellitus (DM), a common chronic non-communicable disease, is on the rise in many countries around the world^1^. Hyperglycemia, insulin resistance, and insulin deficiency are the main characteristics of T2DM, a major type of DM^2^. T2DM is a complex chronic metabolic disease. Besides, blood glucose control, weight control is also important for T2DM patients. Weight loss can lead to reduced insulin resistance and can provide more cardiovascular benefits, while weight gain can further increase insulin resistance, affecting blood sugar control and blood pressure stability^3–5^. Therefore, the choice of more effective and safe drugs assumes special significance for the treatment of T2DM. Of late, in order to develop safe and efficacious drugs for the treatment of T2DM, more and more researchers have focused on GLP-1 drugs represented by gastrointestinal hormone polypeptide drugs, such as semaglutide, exenatide, dulaglutide, albiglutide, etc. ^6–9^. GLP-1 is produced by the enteroendocrine L cells of the intestine to promote insulin release. L cells are distributed at the end of the human gut, esp. in the jejunum, ileum, and colon. GLP-1 can promote insulin secretion or inhibit glucagon production according to the level of blood sugar in vivo. Moreover, it can inhibit appetite and reduce food intake by inhibiting gastric emptying and increasing satiety and may increase β cell regeneration and inhibit β cell apoptosis^10^. Therefore, the clinical application of GLP-1 and similar drugs has created a breakthrough in T2DM treatment.

Dulaglutide is a GLP-1 receptor agonist/drug. A comparison of real-world data done by Chang et al. showed that both dulaglutide and liraglutide have good blood glucose control ability in T2DM patients and have similar effects on weight, blood pressure, liver, and kidney function^11^. Dulaglutide was approved for treatment in China in 2019, and several meta-analyses have reported that dulaglutide showed good efficacy and safety in the treatment of T2DM patients^12–14^. However, most of the T2DM patients included in these meta-analyses hailed from Europe and America, while the Asians were less involved. At the same time, ethnic differences might affect the metabolism of drugs in vivo; thus, affecting the efficacy and safety of drugs^15^. Factors of racial differences include both internal factors such as genetics and physiology (e.g., gene polymorphisms (SNPs), receptor sensitivity, weight, liver, and kidney function, etc.) and external factors such as social culture and living environment (e.g., climate, sunshine, medical measures, eating habits, socioeconomic status, educational status, etc.). Therefore, the racial difference has a great impact on the efficacy and safety of the drug, so whether dulaglutide is effective and safe for T2DM patients in Asia remains poorly understood. To demonstrate the efficacy and safety of dulaglutide in Asian T2DM patients, this study was used to assess the efficacy and safety of dulaglutide compared with other first-line hypoglycemic drugs in the treatment of such patients by meta-analysis.

## Methods

### Data sources and search strategy

We systematically searched Cochrane Library, PubMed, and Embase databases from inception to September 27, 2022, and the language chosen was only English. Unpublished data are available at www.Clinicaltrials.gov. Search terms included “dulaglutide”, “LY2189265”, “Trulicity”, “Type 2 Diabetes Mellitus”, “T2DM”, “Asia”, and “Asian”. The retrieval strategy was adopted by the combination of subject words and free words. Search strategies form all database are as follows: Cochrane Library: (dulaglutide OR LY2189265 OR Trulicity) AND ('Type 2 Diabetes Mellitus' OR T2DM) AND (Asia OR Asian OR China OR Japan). PubMed: ("dulaglutide" [Supplementary Concept] OR LY2189265) AND ('Type 2 Diabetes Mellitus' OR T2DM) AND (Asia OR Asian OR China OR Japan). Embase: ('dulaglutide'/exp OR dulaglutide OR 'ly2189265'/exp OR ly2189265 OR 'trulicity'/exp OR trulicity) AND ('type 2 diabetes mellitus'/exp OR 'type 2 diabetes mellitus' OR 't2dm'/exp OR t2dm) AND ('asia'/exp OR asia OR 'asian'/exp OR asian OR 'china'/exp OR china OR 'japan'/exp OR japan).

According to the inclusion and exclusion criteria, two researchers independently read the title and abstract of the literature for preliminary screening and also read the full text of literature that potentially met the inclusion criteria. Any disagreement was discussed and decided by the third researcher.

### Outcome indicators

The primary outcome indicators included HbA1c, FBG, body weight, and the incidence of adverse events. The secondary outcome indicators included systolic blood pressure, diastolic blood pressure, the incidence of serious adverse events, the incidence of hypoglycemic episodes, and other adverse events. It should be noted that adverse events and serious adverse events are two different concepts^16,17^.

### Study selection and data extraction

The included studies for this meta-analysis met the following criteria: (1) patients aged > 18 years; (2) study site was limited to the Asian country or region; (3) studies containing at least two treatment groups of dulaglutide and other first-line hypoglycemic drugs; (4) studies were RCT or observational study; (5) duration: follow-up time ≥ 10 weeks; (6) documentary language: only English.

Following exclusion criteria were employed: (1) If the study was conducted in a non-Asian country or region; (2) if the control group was a non-first-line hypoglycemic drug; (3) combined use of drugs was reported; (4) incomplete or insufficient data of studies; (5) relevant literature was not available; (6) prospective observational cohort studies; (7) single-arm clinical trials; (8) self-control study; (9) reviews and case reports, and (10) none English literature.

### Data extraction and the risk of bias assessment

Two investigators (MRW and HN) independently extracted the data. When two researchers have different opinions on the same document, they shall be resolved through negotiation through discussion or intervention of the third reviewer (YYR). The characteristics and outcomes were extracted from the text, tables, and figures in studies. The extracted data included basic characteristics of the study, the baseline situation of the patients, the intervention measures, the background treatment, the course of treatment, the outcome index, etc.

We used the Cochrane Collaboration bias assessment tool to evaluate the risk bias of the included RCT studies^18^. This tool evaluates research quality based on the following criteria: (a) Random sequence generation; (b) Allocation concealment; (c) Blinding of participants and personnel; (d) Blinding of outcome assessment; (e) Incomplete outcome data; (f) Selective reporting; and (g) Other biases. The judgment was categorized as “high risk”, “low risk”, or “unclear”. In addition, Newcastle Ottawa scale (NOS) was used to evaluate the quality of the rest document^19^. The evaluation criteria include the selection of study subjects, comparability between groups, and exposure/outcome evaluation^19^. The total score is 9 points, and a score greater than or equal to 5 points indicates that the quality of the literature is qualified. The research design and process of this manuscript are conducted according to PRISMA checklist.

### Publication bias

Funnel plots were used to assess the primary endpoints of potential publication biases^20^. Relative symmetry of individual study estimates was assessed around overall estimates, followed by the Beggs’ tests. At *P* > 0.05, we considered there was no risk of publication bias.

### Statistical analysis

We used Review Manager (RevMan 5.4) software to perform statistical analysis. The weighted mean difference (WMD) was used as the effect analysis statistic for continuous measurement data; risk ratio (RR) was used as the effect analysis statistic for dichotomous variables, and 95%CI was considered for each effect. Statistical heterogeneity between the results was analyzed by Chi-square (χ^2^) test (α = 0.1), and the heterogeneity was quantitatively judged by I^2^. When I^2^ ≤ 50% and *P* > 0.1, the fixed effect model was applied, and when I^2^ > 50% and *P* < 0.1, the random effect model was applied. Additionally, we also investigated the source of heterogeneity with a sensitivity analysis when I^2^ was higher than 50%. The meta-analysis level was set as α = 0.05.

### Patient and public involvement

Patients and/or the public were not involved in the design, or conduct, or reporting, or dissemination plans of this research.

## Results

### Searching results and study characteristics

**Figure 1:**
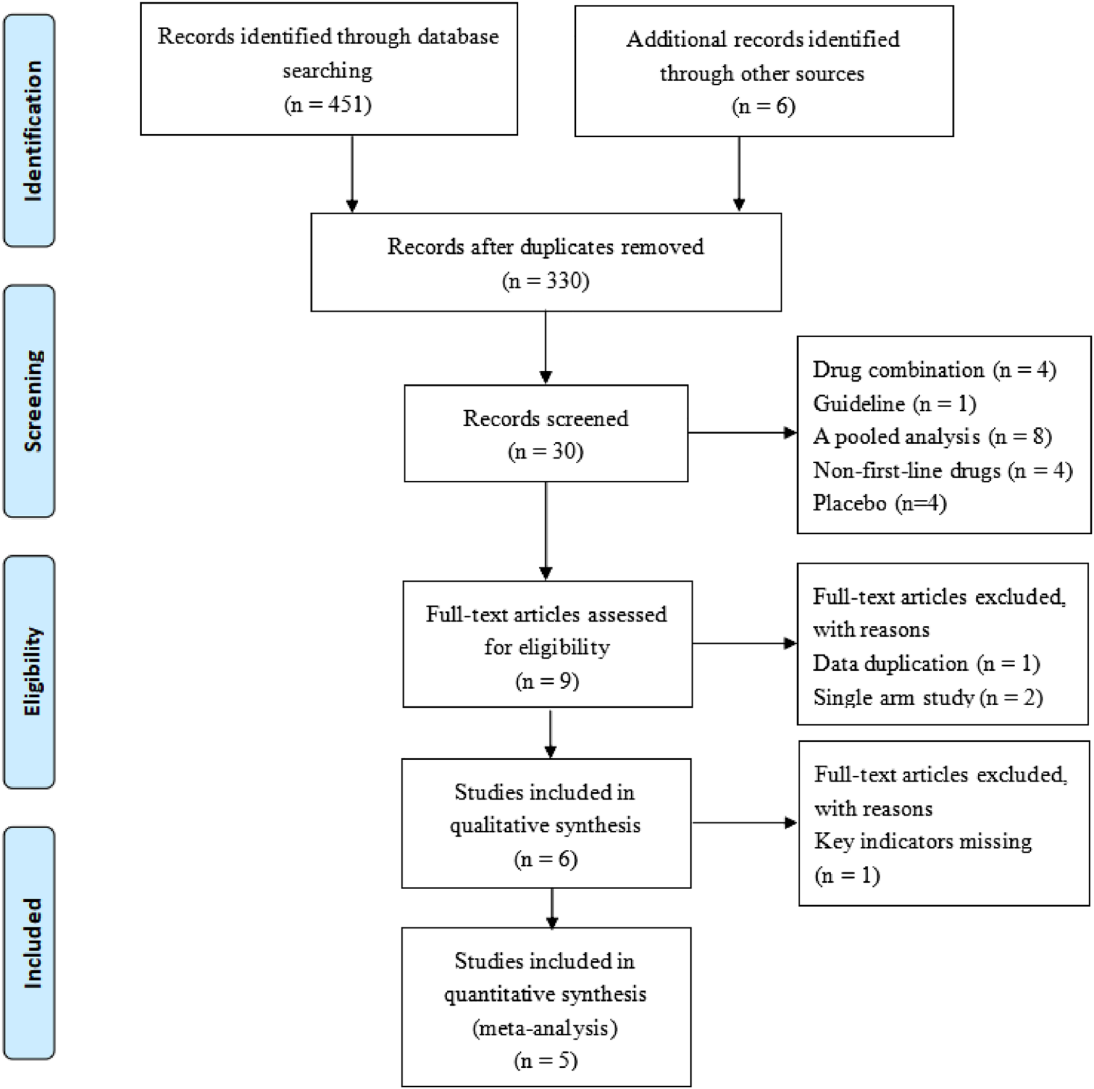
Flow diagram of studies searched in this meta-analysis.

The initial 457 articles were searched, and the duplicate literature was first removed with EndNote software, then the literature was further read for screening, and finally, the 5 studies that conformed to inclusion criteria were included^21–25^. Of them, four were RCTs, and one was an observational retrospective study. A total of 2344 Asian T2DM patients, mainly from Asian countries and regions, including China, Japan, India, Korea, and Taiwan, were involved. Dulaglutide has two doses (0.75 mg and 1.5 mg/subcutaneous injection and once a week), the first-line hypoglycemic drugs included Liraglutide (0.9 or 1.2 mg/day, oral), Glimepiride (1 or 3 mg/day, oral), and Insulin Glargine (subcutaneous injection, once daily). Meanwhile, the duration of intervention in 3 studies^21,22,26^ was 26 weeks. One study had two follow-up cycles (26 weeks and 52 weeks), and another’s duration was 13 weeks. All the studies were published from 2015 to 2020. The literature screening process and results are shown in Fig. 1. Table 1 depicts the basic characteristics of the selected studies.

**Table 1:** General characteristics of included studies.

Author (years)	Participants (n)	Intervention	Dose	Age (years)	BMI (Kg/m^2^)	HbA1c (%)	Weight (kg)	Country	Duration
Miyagawal^21^ 2015	280	Dulaglutide	0.75 mg/week	57.2 ± 9.6	25.6 ± 3.6	8.2 ± 0.8	71.3 ± 12.5	Japan	26 weeks
	137	Liraglutide	0.9 mg/day	57.9 ± 10.4	25.5 ± 3.5	8.1 ± 0.9	70.2 ± 12.5		
Chen^22^ 2018	239	Dulaglutide	0.75 mg/week	53.8 ± 10.1	26.2 ± 3.5	8.0 ± 1.0	–	China, Korean, Taiwan	26 weeks
	239	Dulaglutide	1.5 mg/week	52.7 ± 10.8	25.8 ± 3.4	8.0 ± 1.0	–		
	242	Glimepiride	1–3 mg/day	52.0 ± 10.1	25.7 ± 3.1	7.9 ± 1.0	–		
Shi^23^ 2020	186	Dulaglutide	0.75 mg/week	53.8 ± 9.8	26.0 ± 3.3	8.0 ± 1.0	70.7 ± 12.0	China	26 weeks
	184	Dulaglutide	1.5 mg/week	52.8 ± 10.4	25.5 ± 3.2	8.0 ± 1.0	69.7 ± 10.8		
	186	Glimepiride	1–3 mg/day	52.7 ± 9.6	25.3 ± 2.9	7.9 ± 1.0	69.1 ± 10.6		
Li^24^ 2019	196	Dulaglutide	0.75 mg/week	54.1 ± 10.0	26.2 ± 3.3	8.3 ± 1.1	73.2 ± 12.0	China	26 weeks and 52 weeks
	200	Dulaglutide	1.5 mg/week	54.5 ± 10.0	25.8 ± 3.2	8.4 ± 1.2	71.9 ± 12.2		
	195	Insulin Glargine	Once-daily	55.0 ± 9.2	26.0 ± 3.2	8.3 ± 1.0	72.5 ± 12.5		
Ghosal^25^ 2018	30	Dulaglutide	1.5 mg/week	48.2 ± 8.5	32.8 ± 5.6	8.3 ± 1.2	87.2 ± 11.8	India	13 weeks
	30	Liraglutide	1.2 mg/day	47.3 ± 10.5	34.9 ± 4.7	8.5 ± 1.4	89.4 ± 14.2		

### Quality assessment

**Figure 2:**
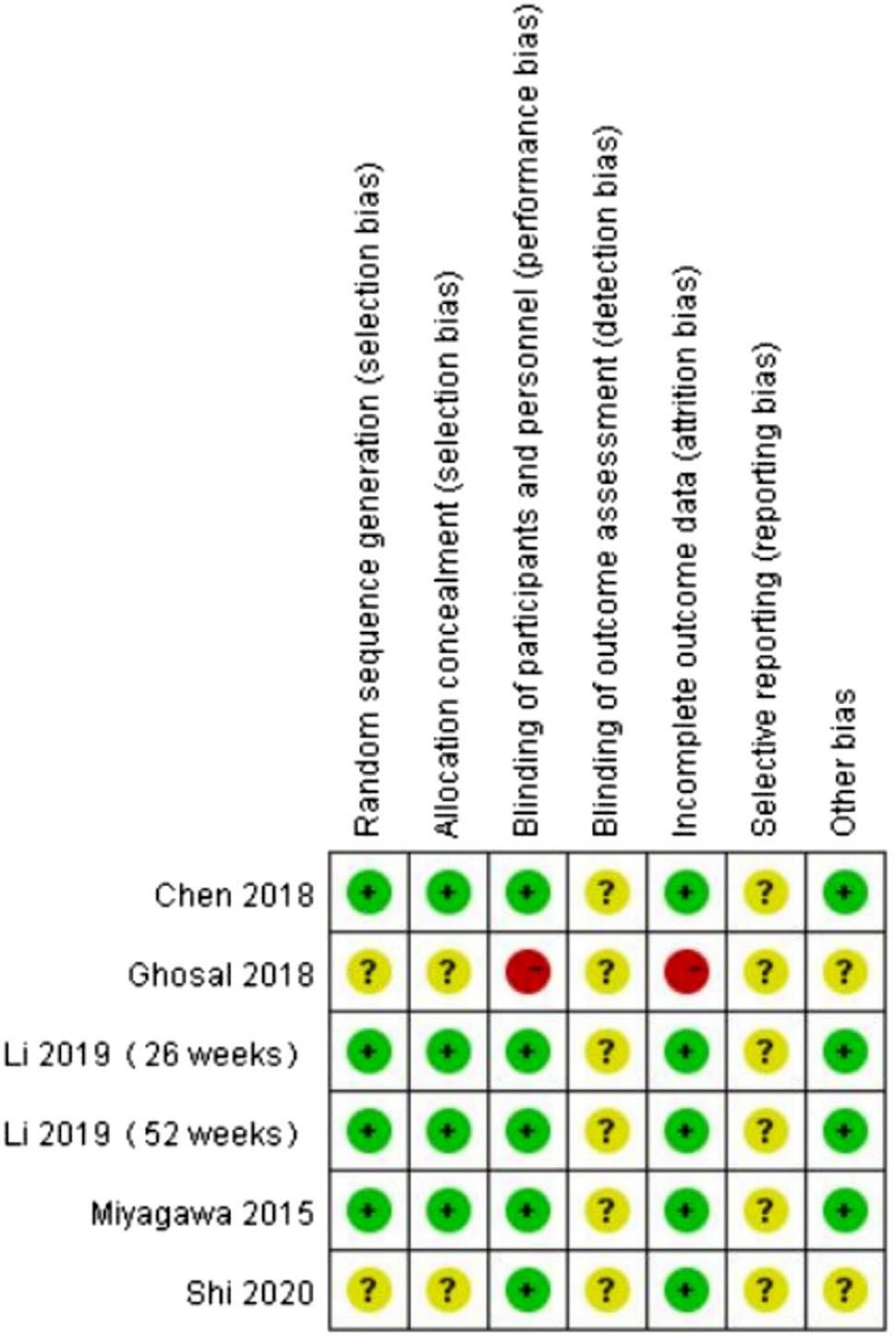
Quality assessment for risk of bias for studies.

**Figure 3:**
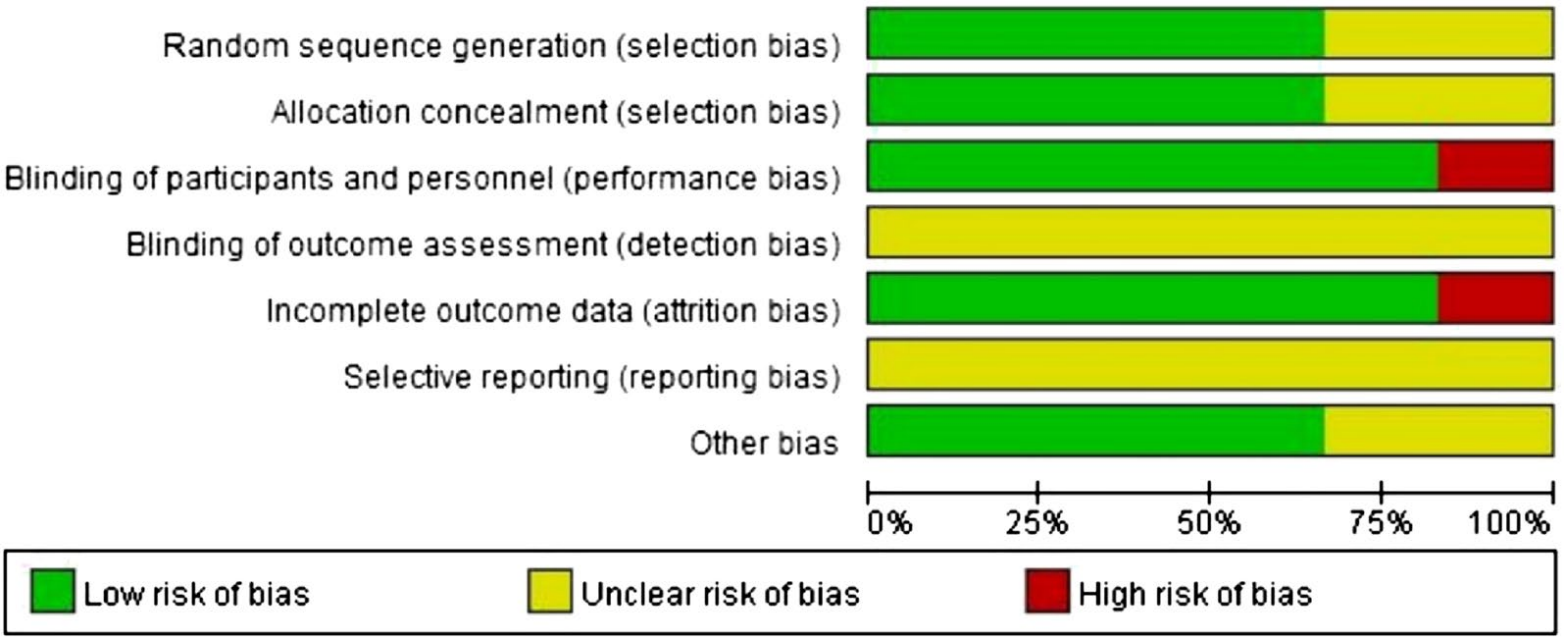
Graphs of risk of bias for studies.

The results of the quality assessment of 5 studies are furnished in Fig. 2. Four studies were RCTs^21–24^ with good quality; one study was an observational retrospective study but of poorer quality than others. Moreover, 3 RCTs ^21,22,24^ described the detailed randomization methods, allocation concealment, blinding of participants and personnel, incomplete outcome data, and other bias. One RCT ^23^ contained the detail of blinding of participants and personnel, and other bias. The relevant information of one study ^25^ was ambiguous. The moderate risks of study design bias are shown in Fig. 3.

### Efficacy analysis

#### HbA1c

**Figure 4:**
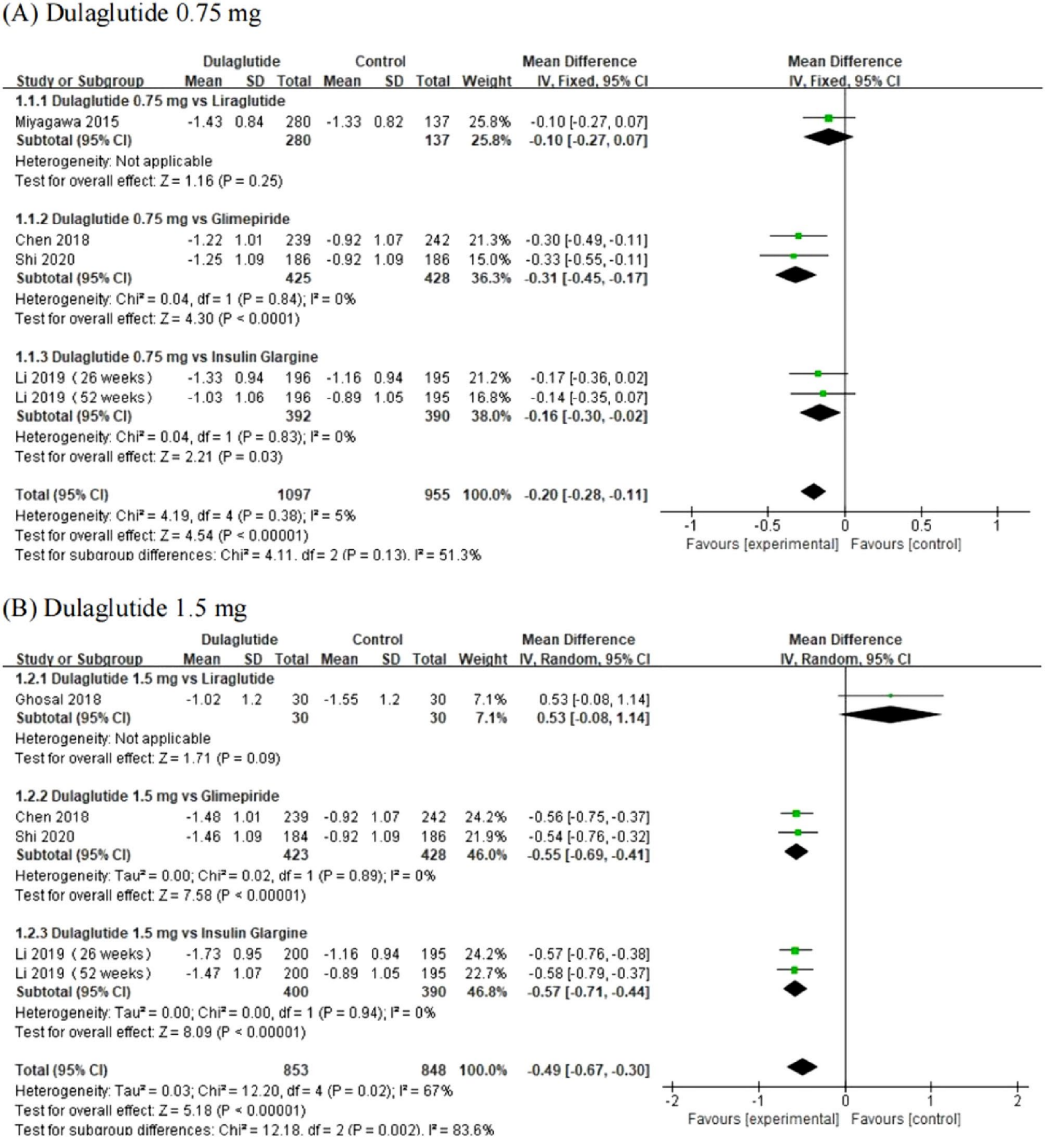
Forest plot of comparing HbA1c between dulaglutide and first-line hypoglycemic drugs.

The changes of HbA1c from baseline between dulaglutide (0.75 mg and 1.5 mg) and first-line hypoglycemic drugs are shown in Fig. 4. Both dose groups of dulaglutide remarkably reduced HbA1c levels [Dulaglutide 0.75 mg: WMD = − 0.20, 95% CI (− 0.28, − 0.11), *P* < 0.0001; Dulaglutide 1.5 mg: WMD = − 0.49, 95% CI (− 0.67, − 0.30), *P* < 0.0001] of patients from Asia. We removed one study ^25^ to ensure a high level of heterogeneity of dulaglutide 1.5 mg group, then the heterogeneity decreased significantly without affecting the overall results [WMD = − 0.56, 95% CI (− 0.66, − 0.46), *P* < 0.0001].

#### FBG

There were no statistically significant differences in FBG [Dulaglutide 0.75 mg: WMD = 0.17, 95% CI (− 0.34, 0.69), *P* = 0.51; Dulaglutide 1.5 mg: WMD = 0.31, 95% CI (− 0.85, 0.24), *P* = 0.27] between dulaglutide (0.75 mg and 1.5 mg) and first-line hypoglycemic drugs.

#### Body weight

Two doses [Dulaglutide 0.75 mg: WMD = − 1.43, 95% CI (− 2.38, − 0.48), *P* = 0.003; Dulaglutide 1.5 mg: WMD = − 2.12, 95% CI (− 2.71, − 1.53), *P* < 0.0001] significantly reduced the body weight of Asian patients, when compared with those of the control groups. Initially, the heterogeneities of two dulaglutide doses were observed high, but when we removed one study^21^ in the dulaglutide 0.75 mg group and one study^25^ in dulaglutide 1.5 mg group, the heterogeneities in both groups decreased remarkably. Moreover, the overall estimate results [Dulaglutide 0.75 mg: WMD = − 1.87, 95% CI (− 2.15, − 1.60), *P* < 0.0001; Dulaglutide 1.5 mg: WMD = − 2.40, 95% CI (− 2.68, − 2.13), *P* < 0.0001] were not affected.

#### Blood pressure

There were no statistically significant differences noted in systolic blood pressure and diastolic blood pressure for the Asian T2DM patients between dulaglutide (0.75 mg and 1.5 mg) and first-line hypoglycemic drugs. The results are shown in Table 2.

**Table 2:** The results of efficacy and safety in meta-analysis.

Outcomes	Subgroup	Studies (n)	Participants	I^2^	Effect estimate	*P* value
HbA1c	Dulaglutide 0.75 mg Dulaglutide 1.5 mg	4 4	2052 2052	5 67	− 0.20 [− 0.28, − 0.11] − 0.49 [− 0.67, − 0.30]	< 0.0001 < 0.0001
FPG	dulaglutide 0.75 mg dulaglutide 1.5 mg	4 4	2052 1701	89 83	0.17 [− 0.34, 0.69] 0.31 [-0.85, 0.24]	0.51 0.27
Body Weight	dulaglutide 0.75 mg dulaglutide 1.5 mg	4 4	2052 1701	94 76	− 1.43 [− 2.38, − 0.48] − 2.12 [− 2.71, − 1.53]	0.003 < 0.0001
Systolic blood pressure	dulaglutide 0.75 mg dulaglutide 1.5 mg	3 3	1680 1331	52 30	0.75 [− 2.39, 3.90] − 0.74 [− 2.88, 1.40]	0.64 0.50
Diastolic blood pressure	dulaglutide 0.75 mg dulaglutide 1.5 mg	3 3	1680 1331	0 0	0.25 [− 0.74, 1.23] − 0.19 [− 1.59, 1.22]	0.62 0.79
HbA1c ≤ 6.5%	dulaglutide 0.75 mg dulaglutide 1.5 mg	4 3	2052 1641	79 84	1.34 [1.05, 1.71] 1.67 [1.21, 2.31]	0.02 0.002
Adverse events	dulaglutide 0.75 mg dulaglutide 1.5 mg	4 3	2052 1641	19 80	1.09 [1.01, 1.18] 1.12 [0.94, 1.35]	0.02 0.21
Serious adverse events	dulaglutide 0.75 mg dulaglutide 1.5 mg	4 3	2052 1641	0 0	1.35 [0.77, 2.36] 2.03 [1.17, 3.53]	0.29 0.01
Hypoglycemic episodes	dulaglutide 0.75 mg dulaglutide 1.5 mg	4 3	2052 1641	90 89	0.35 [0.16, 0.75] 0.35 [0.18, 0.67]	0.007 0.002
Nasopharyngitis	dulaglutide 0.75 mg dulaglutide 1.5 mg	3 2	1270 851	0 0	1.09 [0.74, 1.61] 1.19 [0.69, 2.04]	0.67 0.53
Decreased appetite	dulaglutide 0.75 mg dulaglutide 1.5 mg	4 3	2052 11,641	84 0	9.66 [2.18, 42.82] 21.56 [6.79, 68.50]	0.16 < 0.0001
Diarrhoea	dulaglutide 0.75 mg dulaglutide 1.5 mg	4 3	2052 1641	0 0	2.63 [1.78, 3.88] 5.16 [3.46, 7.69]	< 0.0001 < 0.0001
Nausea	dulaglutide 0.75 mg dulaglutide 1.5 mg	4 4	2052 1701	73 83	3.31 [0.92, 11.91] 7.20 [1.44, 36.09]	0.07 0.02
Abdominal distension	dulaglutide 0.75 mg dulaglutide 1.5 mg	4 4	2052 1641	73 50	2.11 [0.52, 8.62] 6.80 [3.51, 13.16]	0.30 < 0.0001
Renal and urinary disorders	dulaglutide 0.75 mg dulaglutide 1.5 mg	3 2	1289 876	0 0	1.01 [0.59, 1.70] 1.14 [0.63, 2.07]	0.98 0.66
Psychiatric disorders	dulaglutide 0.75 mg dulaglutide 1.5 mg	3 2	1289 876	0 0	0.39 [0.13, 1.15] 1.15 [0.42, 3.13]	0.09 0.79
Eye disorders	dulaglutide 0.75 mg dulaglutide 1.5 mg	3 2	1289 876	45 71	0.65 [0.31, 1.37] 0.81 [0.35, 1.91]	0.26 0.63
Cardiac disorders	dulaglutide 0.75 mg dulaglutide 1.5 mg	3 2	1289 876	0 88	1.36 [0.76, 2.43] 0.75 [0.07, 8.32]	0.29 0.81
Endocrine disorders	dulaglutide 0.75 mg dulaglutide 1.5 mg	3 2	1289 876	0 0	1.73 [0.37, 8.02 2.17 [0.49, 9.59]	0.48 0.31
Nervous system disorders	dulaglutide 0.75 mg dulaglutide 1.5 mg	3 2	1289 876	0 0	1.04 [0.70, 1.53] 1.32 [0.82, 2.13]	0.86 0.26
Reproductive system disorders	dulaglutide 0.75 mg dulaglutide 1.5 mg	3 2	1289 876	20 0	4.06 [1.29, 12.80] 4.97 [0.58, 42.35]	0.02 0.14
Neoplasms	dulaglutide 0.75 mg dulaglutide 1.5 mg	3 2	1289 876	59 0	0.97 [0.07, 13.99] 5.93 [0.71, 49.31]	0.98 0.26

### Safety analysis

#### Adverse events

**Figure 5:**
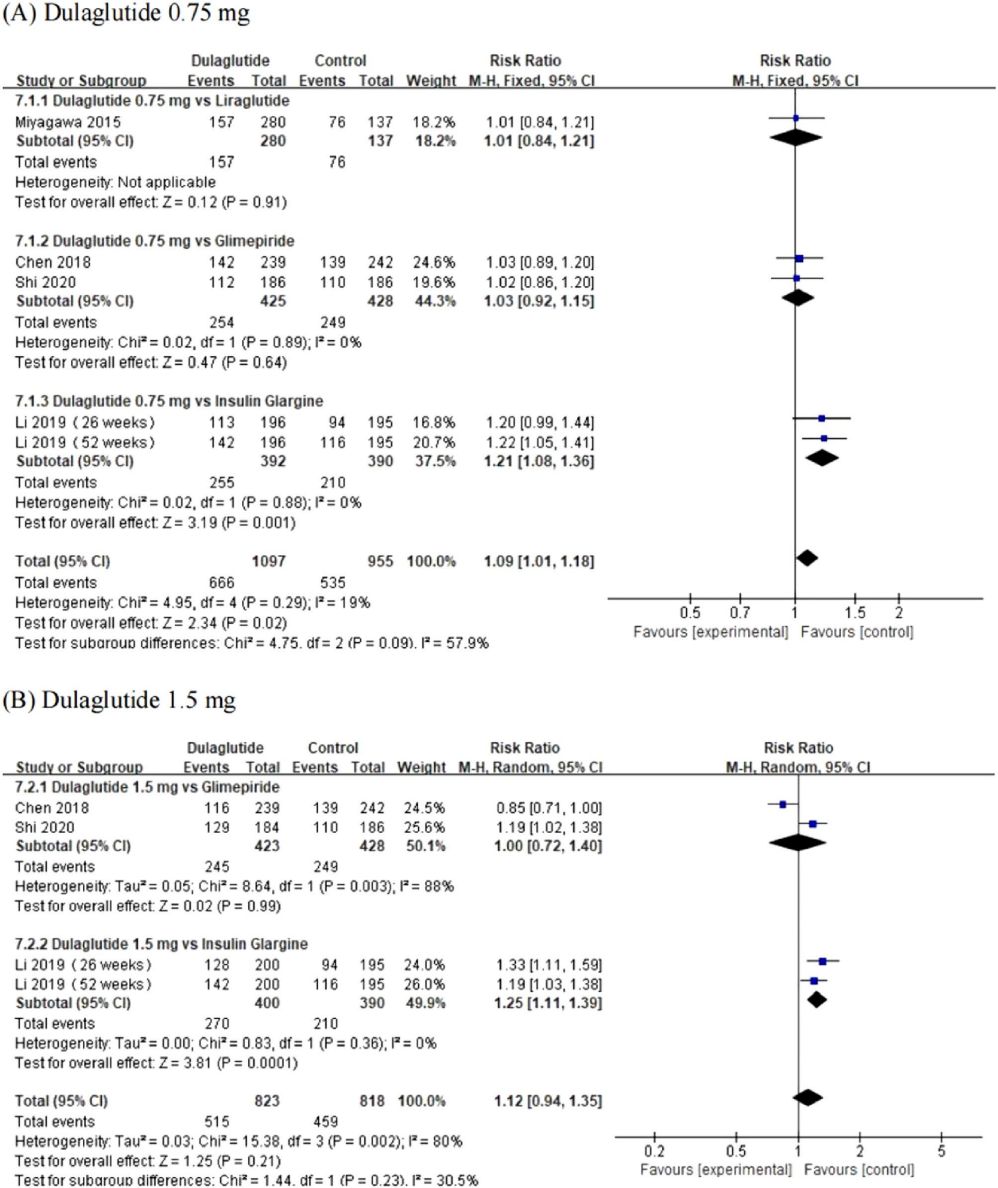
Forest plot of comparing adverse events between dulaglutide and first-line hypoglycemic drugs.

The incidence of adverse events [RR = 1.09, 95% CI (1.01, − 1.18), *P* = 0.02] in the dulaglutide 0.75 mg group was slightly higher than that in first-line hypoglycemic drugs. However, there was no statistically significant difference in the incidence of adverse events [RR = 1.12, 95% CI (0.94, − 1.35), *P* = 0.21] in the dulaglutide1.5 mg group compared with first-line hypoglycemic drugs. The change of the incidence of adverse events from baseline between dulaglutide (0.75 mg and 1.5 mg) and first-line hypoglycemic drugs are shown in Fig. 5.

#### Serious adverse events

The difference was statistically non-significant in the incidence of serious adverse events [RR = 1.35, 95% CI (0.77, 2.36), *P* = 0.29] between dulaglutide 0.75 mg, and the control groups. The incidence of adverse events [RR = 2.03, 95% CI (1.17, 3.53), *P* = 0.01] in the dulaglutide 1.5 mg group was slightly higher than that in first-line hypoglycemic drugs.

### Hypoglycemic episodes

The incidences of hypoglycemic episodes in both dulaglutide (0.75 mg and 1.5 mg) groups were higher than that in first-line hypoglycemic drugs. The results are shown in Table 2.

### Other adverse events

After consuming dulaglutide, most of the patients experienced a loss of appetite, diarrhea, nausea, and abdominal distension. When compared with the first-line hypoglycemic drugs, the dulaglutide 1.5 mg group showed a greater incidence of loss of appetite, diarrhea, nausea, and abdominal distension, and the dulaglutide 0.75 mg group had no significant difference in these three aspects. Meanwhile, there were no statistically significant differences in the incidence of renal and urinary disorders, psychiatric disorders, eye disorders, cardiac disorders, endocrine disorders, nervous system disorders, and neoplasms between dulaglutide (0.75 mg and 1.5 mg) and first-line hypoglycemic drugs. Besides, the dulaglutide 0.75 mg group showed a higher incidence of reproductive system disorders, but no difference in the dulaglutide 1.5 mg group and control group was noticed (Table 2).

### Publication bias

**Figure 6:**
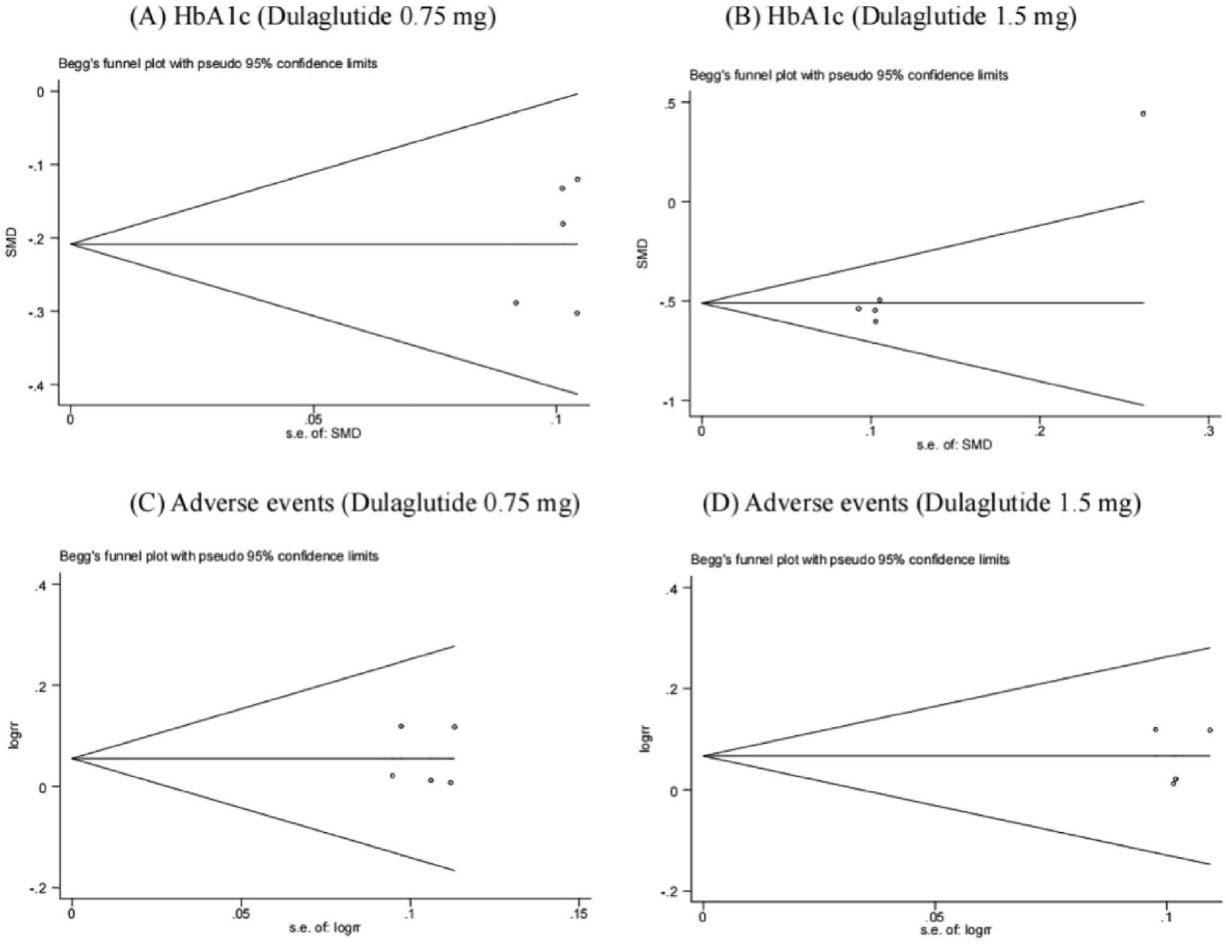
Publication bias of primary outcome indications.

Stata 12.0 software was used for publication bias analysis of levels of HbA1c, FBG, bodyweight, and adverse events in patients after consumption of the drug. The Beggs’ tests did not find significant publication bias across the studies (*P* = 0.806, P = 0.462, P = 0.807, P = 0.991, respectively). The results are shown in Fig. 6.

## Discussions

In this meta-analysis, a systematic review to assess the efficacy and safety of dulaglutide in Asian T2DM patients was conducted. Both doses of dulaglutide (0.75 mg and 1.5 mg) were more effective than other first-line hypoglycemic drugs (Liraglutide, Glimepiride, Insulin Glargine) in reducing HbA1c and bodyweight levels. However, the effect of both doses of dulaglutide groups in reducing FBG seems the same when compared with other first-line drugs. Meanwhile, the effect of two dulaglutide groups and first-line drugs was similar in reducing the blood pressure (systolic blood pressure and diastolic blood pressure). Also, the percentage of participants who achieved HbA1c ≤ 6.5% was improved in dulaglutide dose groups showing its better efficacy than in the control groups.

Previous meta-analyses reported that dulaglutide was effective and safe for T2DM patients^12–14,27^. But most of the analyses included T2DM patients (of the White, American Indian, African American, Black, or Alaska Native races mainly), with a few Asian patients as well. At the same time, the control groups in some studies^12,27^ were placebo. Although three studies^13,14,27^ had reported multiple-dose groups of dulaglutide, some countries or regions in Asia, such as China, just approved only two dose groups of dulaglutide for marketing (only 0.75 mg and 1.5 mg). Furthermore, one study^12^ included 3 RCTs related to dulaglutide with one dose group, and one study^13^ included 2 RTCs of dulaglutide. Network meta-analysis results by Webbs et al.^14^ showed that semaglutide (0.5 mg) provided greater efficacy than dulaglutide (0.75 mg), but it included four trials of semaglutide and one trial of dulaglutide, and there were no safety results; hence, the results need to be further confirmed. Unlike previous systematic reviews, we performed meta-analyses of the efficacy and safety of dulaglutide in Asian T2DM patients to further estimate the efficacy and safety of dulaglutide. We included four trials and one retrospective cohort study that were published from 2015 to 2020 and 2 doses of dulaglutide (0.75 mg and 1.5 mg) compared with other first-line hypoglycemic drugs. At the same time, we also performed a heterogeneity analysis and publication bias in this study.

We analyzed dulaglutide in two dose groups separately; 0.75 mg dulaglutide could significantly reduce the HbA1c, body weight and had the same efficacy in FBG compared with the control group. For body weight, we also analyzed the source of heterogeneity. Ghosal ^25^ noticed an increase in heterogeneity when the source of heterogeneity was removed, but the overall result was not affected with lower heterogeneity. In the 1.5 mg dulaglutide group, it also significantly reduced the HbA1c, body weight, and had similar efficacy in FBG. Higher heterogeneity in HbA1c and body weight was observed, and the reasons for the heterogeneity were analyzed. The overall result was not affected, and the heterogeneity decreased. Two dulaglutide groups could reduce HbA1c by ≤ 6.5% in more patients. As regards blood pressure, dulaglutide showed similar efficacy as with the control group. Our study indicated better efficacy for Asian T2DM patients by two doses of dulaglutide than compared to first-line drugs. In terms of safety, 0.75 mg dulaglutide had a higher incidence of adverse events than the control group, but 1.5 mg showed no remarkable difference in the incidence of adverse events. Most of the Asian patients developed gastrointestinal discomfort, as observed in the European or American trials^28–31^, including loss of appetite, diarrhea, nausea, and abdominal distension, and both dose groups showed a higher incidence of diarrhea than the control group. Hypoglycemia is an inevitable adverse reaction in the hypoglycemic treatment of diabetic patients and is a limiting factor for the long-term maintenance of normal blood glucose levels in diabetic patients ^32^. Therefore, we should pay attention to the risk of hypoglycemia when choosing hypoglycemic drugs. We found that both doses of dulaglutide were associated with an increased risk of hypoglycemic events. Interestingly, this result differed from that of Singhal’s study ^13^, which indicated that Asian T2DM patients should pay attention to their blood sugar control and show alertness to the occurrence of dizziness, pallor, sweating, palpitation, and other conditions. Additionally, we also assessed the incidence of other adverse events in dulaglutide compared with first-line drugs (Table 2).

Odawara^33^ reported that nasopharyngitis was also a common adverse event in the process of using dulaglutide, so we supplemented the results in this study. Dulaglutide (0.75 mg and 1.5 mg) was comparable to the control group in the incidence of other adverse events and did not increase the incidence of other adverse events, except for 0.75 mg dulaglutide in reproductive system disorders, which was marginally higher than the control group. Overall, the safety of dulaglutide was generally good, but patients may develop contraindications of hypoglycemia and gastrointestinal discomfort.

### Limitations

There are limitations in the current study. First, all articles were published in English; there may exist some potential risks because some papers were published in other languages, and they could not be included. Second, the follow-up time of the included studies was inconsistent; four trials lasted 26 weeks, one lasted 52 weeks, and one trial lasted 13 weeks. The difference in follow-up time may affect the efficacy and safety of patients. Third, one study had limited data for further analysis, and it might have impacted the outcome measures. More research is suggested on available guidance.

## Conclusion

To sum up, this meta-analysis indicated that dulaglutide (0.75 mg and 1.5 mg) could significantly decrease HbA1c and body weight in Asian T2DM patients and also has similar efficacy in decreasing FBG when compared with first-line hypoglycemic drugs. However, Asian T2DM patients are suggested to notice the occurrence of hypoglycemia and gastrointestinal discomfort.

## Data Availability

All the data supporting this systematic review are from previously reported studies and data sets, which have been cited. The processed data are available from the corresponding author upon request.
